# Evaluation of the performance of radiologists assisted by AI in detecting colorectal liver metastases on contrast-enhanced CT

**DOI:** 10.1186/s40644-026-00998-x

**Published:** 2026-02-16

**Authors:** Jeong Hee Yoon, Hyo-Jin Kang, Jae Seok Bae, Jae Hyun Kim, Jin Sol Choi, Won Hyeong Im, Alexandre Bône, Jeong Min Lee

**Affiliations:** 1 Department of Radiology, Seoul National University Hospital, Seoul, 03080 Republic of Korea; 2 Department of Radiology, Seoul National University College of Medicine, Seoul, 03080 Republic of Korea; 3 Department of Radiology, Presbyterian Medical Center, Jeonju, Republic of Korea; 4 Guerbet Research, Villepinte, France; 5 Institute of Radiation Medicine, Seoul National University Medical Research Center, Seoul, Republic of Korea

**Keywords:** Colorectal neoplasms, Liver metastasis, Computed tomography, Artificial intelligence, Computer-assisted diagnosis, Radiologist performance, Diagnostic accuracy, Workflow efficiency

## Abstract

**Background:**

Colorectal liver metastasis (CRLM) detection on contrast-enhanced CT (CECT) remains challenging due to low tumor-to-liver contrast. This study aimed to evaluate the performance of 2.5D U-net based artificial intelligence (AI) software for focal liver lesion (FLL) detection on CECT and its added value by comparing radiologists’ performance with and without AI support.

**Methods:**

This retrospective study included patients with colorectal cancer between January 2008 and December 2011, with available preoperative CECT. Six radiologists consisting of three attendings and three fellows read the CECT images in four review sessions: reporting all FLLs and only suspicious colorectal liver metastasis (CRLM), both with and without AI. The detection rates of FLL and CRLM, diagnostic performance of CRLM, and the reading time were compared between the sessions, using reference standards of pathology, follow-up CECT or gadoxetic acid-enhanced MRI.

**Results:**

The study included 277 patients (median age 70 years, 182 male) with 989 FLLs (median size 6 mm, 324 CRLMs). The figures-of-merit of AI and radiologists were 0.82 (95% CI: 0.77, 0.86) and 0.86 (95% CI: 0.81, 0.89) in FLLs ≥ 10 mm (*P* = 0.188), and 0.53 (95% CI: 0.48, 0.62) and 0.60 (95% CI: 0.56, 0.64) in 5–9 mm FLLs (*P* = 0.145). In sessions reporting only suspicious CRLM, AI assistance increased pooled sensitivity (62.5% [1215/1944] vs. 66.8% [1299/1944], *P* < 0.001) while it maintained pooled specificity (89.6% [3574/3990] vs. 89.8% [3584/3990], *P* = 0.730) in per-lesion analysis. The median reading time decreased with AI when reporting all FLLs from 38.0 s to 30.0 s (*P* < 0.001). With AI assistance, the reading time gap between senior and junior radiologists decreased from 16.0 s to 9.0 s in sessions reporting all FLLs and decreased from 24.0 s to 14.0 s in sessions reporting only suspicious CRLMs.

**Conclusions:**

AI software may improve radiologists’ performance by increasing the sensitivity of diagnosing CRLM on CECT, without decreasing specificity, and reducing the reading time.

**Trial registration:**

Not indicated.

**Supplementary Information:**

The online version contains supplementary material available at 10.1186/s40644-026-00998-x.

## Background

Colorectal cancer (CRC) is the third most common cancer worldwide [1]. Up to 20% of newly diagnosed CRC patients have distant metastases at presentation [2] and 25–50% of CRC patients develop colorectal liver metastasis (CRLM) during follow-up. CRLM significantly impacts patient prognosis, and early detection is crucial for appropriate treatment planning and improved outcomes. Abdominopelvic contrast-enhanced computed tomography (CECT) is recommended for the staging workup and post-treatment surveillance. However, it can be challenging to identify small CRLMs due to the low contrast between CRLM and the background liver on CECT. Attempts have been made to mitigate this issue using artificial intelligence (AI) [3–6]. An automatic tumor segmentation program could help radiologists to reduce the reading time by annotating and precisely segmenting focal liver lesions (FLLs). However, clinical utility of artificial intelligence tools for CRLM detection on CT has not been thoroughly evaluated.

This study aimed to evaluate the performance of AI software for FLL detection on CECT and its added clinical value by comparing radiologists’ performance with and without AI support.

## Methods

### Study design and setting

This retrospective, single-center study was conducted at Seoul National University Hospital to evaluate the performance of 2.5D U-net based AI software for FLL detection on CECT and its added clinical value by comparing radiologists’ performance with and without AI support. The Institutional Review Board of Seoul National University Hospital (No. H-1812-013-991) approved this retrospective study and was performed in accordance with the 1964 Helsinki Declaration and its later amendments, informed consent was waived. This work was funded by Guerbet, which developed the AI software in collaboration with IBM Watson Health. One of the authors (A.B.) is an employee of Guerbet; however, the remaining authors maintained full control over all data and the submission process.

### Patient selection

We searched the radiologic database for patients with surgically diagnosed CRC who underwent CECT for suspected CRLM between January 2008 and December 2011 and had available reference standards for FLLs. The exclusion criteria were (a) prior local therapy for CRLM; (b) absence of adequate portal venous phase (PVP) imaging; (c) inadequate poor image quality (significant motion or prosthesis artifacts, slice thickness > 5 mm); (d) inappropriate reference standards to confirm the number or characteristics of FLLs. The appropriate reference standard for FLLs was defined as either surgery within 8 weeks of CECT or gadoxetic acid-enhanced liver MRI for both benign lesions and malignancies. Follow-up CECT for more than 6 months was used to verify stability of benign FLLs [7, 8].

### Image acquisition

CECT was performed using 21 different scanners with 2–320 channels at 120–140 kVp, and tube-current modulation was applied in available scanners. The PVP was obtained 70–80 s after contrast medium administration. Axial images were reconstructed to have a slice thickness of 3–5 mm with filtered back-projection or vendor-specific hybrid iterative reconstruction. Detailed information is described in the Supplement.

### AI software development and implementation

*AI model development—* The AI software used in this study (DUOnco for Liver [DUO-L]; Guerbet) was developed using a 2.5D U-Net architecture. An AI model was developed using a database of 4580 abdominal CECT studies collected from multiple centers across North America, Europe, and Asia. Volumetric segmentation was performed for all visible FLLs by one of 32 radiologists with at least 3 years of experience in reading abdominal CECT. The main algorithmic module of the model consisted of an ensemble of U-Nets trained to segment FLLs in axial portal venous phase series [9]. The image processing pipeline also included several algorithmic modules dedicated to identifying the body part, liver presence, and the contrast phase, as well as liver segmentation, FLL segmentation refinement, false positive detection, and lesion measurement. A sensitivity of 87.4% [95% CI: 85.6, 89.1] for FLL detection was found for the AI model in internal testing with FLLs ≥ 10 mm. Details about AI model development in Supplement, Table S1 and Fig. S1.

**Fig. 1:**
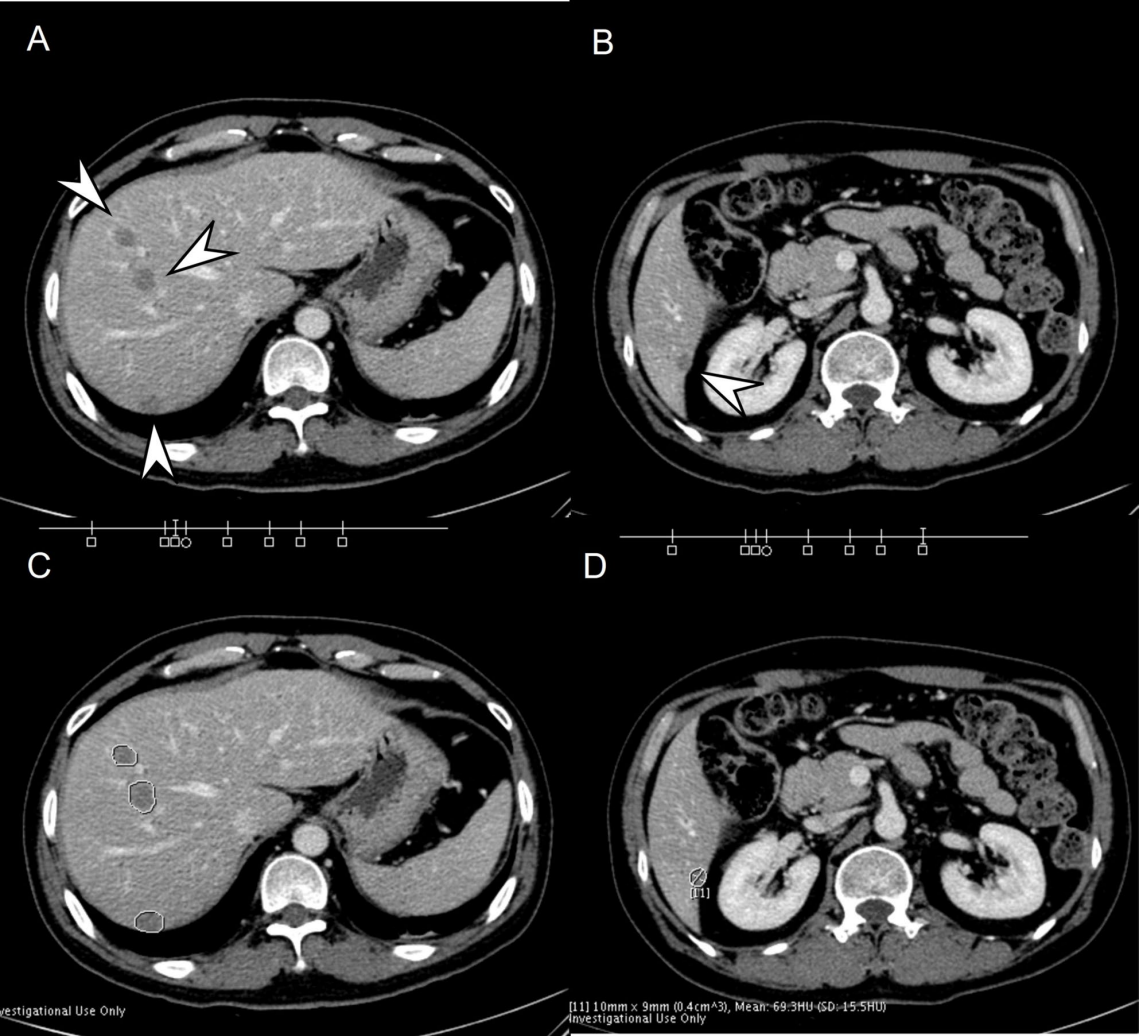
A 39-year-old male patient with surgically confirmed colorectal liver metastases (CRLMs). On portal venous phase (**A**, **B**), multiple CRLMs are seen in the bilateral hemilivers (arrowheads). All seen metastases are detected and correctly segmented with the artificial intelligence (AI) software (**C**, **D**). On the AI software processed images (**C**, **D**), the round and square shapes of the bar indicate the location of the segmented focal liver lesions (FLLs) and its size category (round for FLLs < 10 mm, square for FLLs ≥ 10 mm). The bidimensional size, volume, and Hounsfield unit (HU) of FLL are also reported in the order of segmentation from the craniocaudal direction. The segmented CRLM in segment 6 is 10 × 9 mm, mean HU is 69.3 and it is 11th detected FLL in this patient (**D**)

*AI software—* The FLL detection and segmentation software based on the developed AI model (DUOnco for Liver [DUO-L]; Guerbet) was used to process the images and visualize the results. The software was installed on the console within the hospital connecting to an independent, closed-server system. No specific image processing was performed, and anonymized data was then processed by a trained research assistant. Failure cases were checked by the principal investigator (J.M.L., with 25 years of clinical experience) to identify the cause of failure. The processed image showed the segmented FLLs with the size, volume, and mean Hounsfield units, and the location of the segmented FLLs was presented in the upper quadrant of the CT images. Depending on the FLL size, round (< 10 mm) or square (≥ 10 mm) shapes were presented (Fig. 1). If no FLLs were detected, the processed image revealed only a single slice image quoting “no focal liver lesion(s) identified.”

### Image analysis

*Reviewers—* Six body radiologists with varying levels of experience were recruited and grouped as senior (*n* = 3, J.H.Y, H.K. and J.S.B. with 12, 7, 6 years of clinical experience after body imaging fellowship) and junior (*n* = 3, J.H.K., J.S.C., and W.H.I. in their fellowship training). All cases were anonymized and randomly distributed to all reviewers in each review session. Reviewers were blinded to any information, except that the study population had colorectal cancer and CECT was taken for metastasis surveillance.

*Review sessions—* Four independent reading sessions were conducted on commercially available DICOM viewer (RadiAnt DICOM Viewer, Medixant) using axial PVP images consistent with the AI software. Sessions 1 and 2 required reviewers to identify all FLLs except calcifications and surgical clips, whereas sessions 3 and 4 focused exclusively on lesions suspicious for CRLM. Sessions 1 and 3 provided only PVP images, while sessions 2 and 4 additionally included software-annotated PVP images. In each session, reviewers recorded the size, location, and likelihood of metastasis on a 5-point scale (1: definitely not metastasis, 2: probably not metastasis, 3: indeterminate, 4: probably metastasis, 5: definitely metastasis). Reading times were measured using a digital stopwatch. Senior reviewers completed sessions 1 and 2 first, and junior reviewers began with sessions 3 and 4. A 4–8-week interval separated the sessions.

### Reference standard

**Fig. 2:**
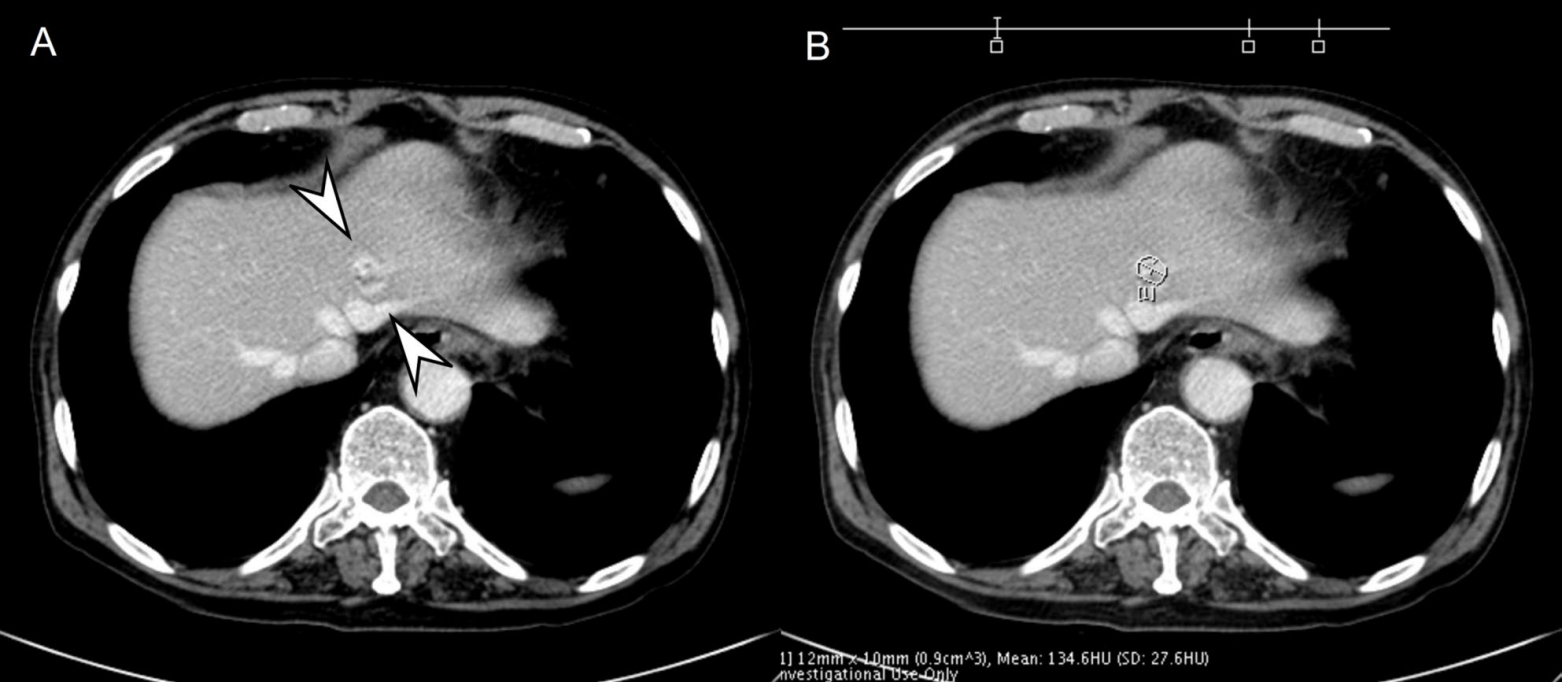
A 79-year-old male patient with clinically diagnosed hemangioma. On portal venous phase (**A**), 18 mm hemangioma is seen in the left lateral segment (arrowheads). On processed image with AI software, hemangioma is partially segmented and measured (**B**). The hemangioma is regarded as being correctly detected but incorrectly segmented by the software

One senior body radiologist (J.M.L.) who did not participate in the image analysis sessions determined FLL presence or absence using the index exam, follow-up studies, and AI-tumor segmentation. Malignant lesions were confirmed with histology within 8 weeks of CECT. Benign FLLs were confirmed with characteristic findings and stability on follow-up imaging. Details on clinical diagnoses and FLL counts are described in the Supplement. Lastly, Segmentation quality was checked visually; if more than 50% of an FLL was unsegmented or if ≥ 50% of the segmented area extended into normal liver or other structures, it was deemed inappropriate (Fig. 2).

### Outcomes measures

The primary outcome was to evaluate radiologists’ performance in detecting FLLs and CRLMs with and without AI assistance. Secondary outcomes included comparisons of (a) standalone AI performance versus radiologists alone; (b) diagnostic accuracy for CRLM with and without AI assistance; (c) reading times with and without AI support; (d) radiologists’ performance across differing reporting scopes (reporting all FLLs versus only suspicious CRLMs) with AI assistance; and (e) radiologists’ performance based on clinical experience levels with and without AI assistance.

### Statistical analysis

The detection rates for all FLLs and CRLMs were assessed with generalized estimating equations (GEEs) and weighted jackknife free-response receiver operating curve (JAFROC) analysis. Correlations between multiple FLLs in a patient were accounted for through a binomial distribution and logit link function. The Figure-of-merit (FOM) in JAFROC analysis, ranging from 0 to 1 and representing the probability that a lesion is rated higher than a non-lesion with higher values indicating better detection performance, was compared between sessions with and without AI assistance. Multi-reader multi-case analysis and GEEs were employed to compare sensitivity, specificity, positive predictive value (PPV), negative predictive value (NPV), and accuracy for diagnosing CRLM between sessions with and without AI assistance. All detection and diagnostic accuracy analyses were performed on a per-lesion basis, and we performed additional per-patient analysis for CRLM diagnosis. A patient-level diagnosis was considered positive when a reader identified at least one lesion as CRLM (confidence score ≥ 3). The effect of AI was assessed using GEE with a binomial distribution, identity link function, and exchangeable correlation structure, accounting for clustering within patient-reader pairs. Reading time was compared between the sessions using the Wilcoxon signed-rank test, and the Hodges-Lehmann estimator with its 95% confidence interval (CI) was reported as a measure of location shift. For independent group comparisons (junior vs. senior radiologists), the Mann-Whitney U test was used, and the Hodges-Lehmann estimator for two samples with bootstrap 95% CI was calculated. As a sensitivity analysis, a linear mixed-effects model was used to assess the interaction between AI assistance and experience level, with reader as a random effect. SPSS (version 26, IBM), SAS (version 9.4; SAS Institute inc.), Python 3.12 with scipy and statsmodels packages and R software (version 4.0.3; R package – MRMCaov, RJafroc) were used.

## Results

**Fig. 3:**
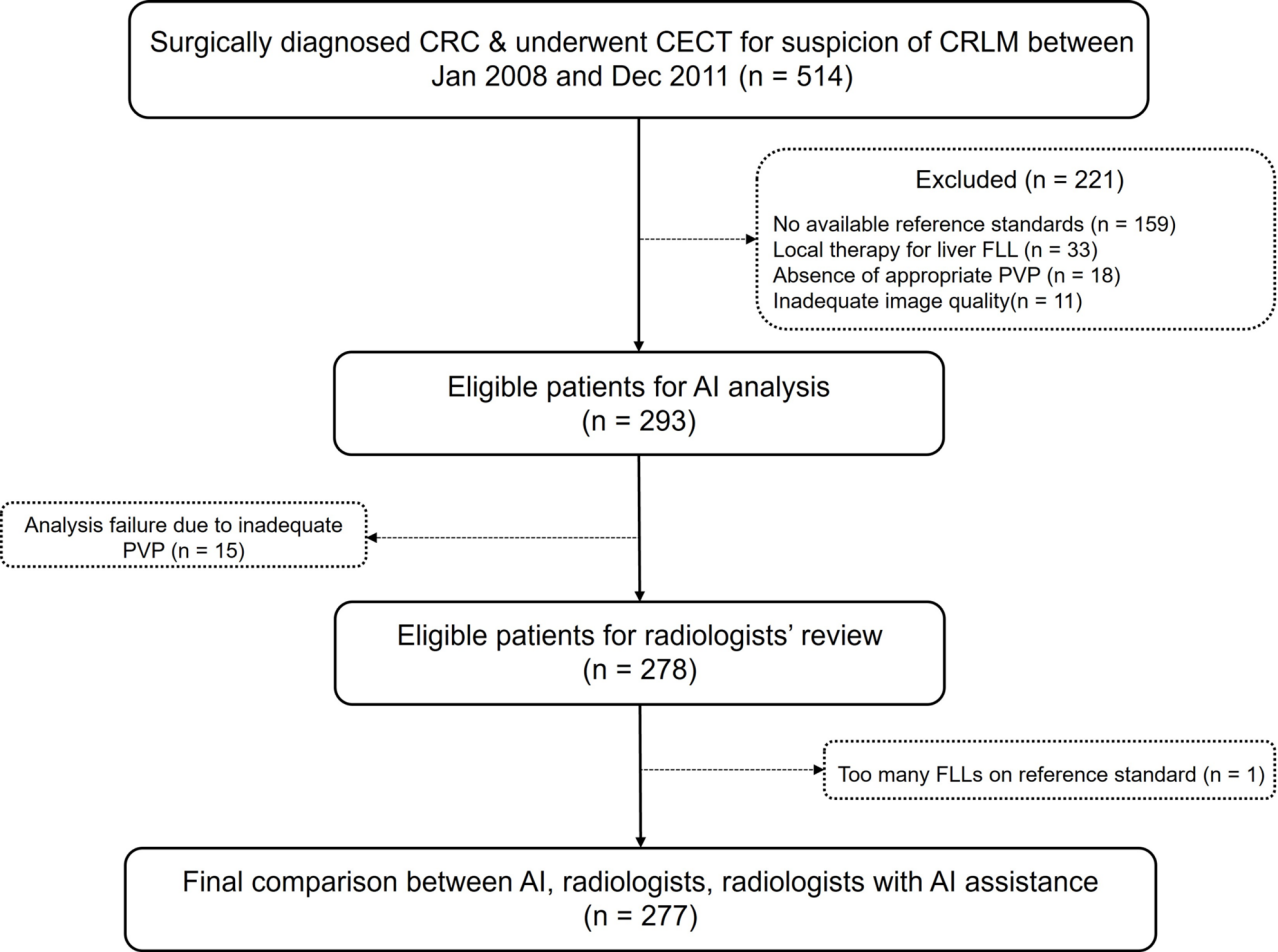
Study flow. AI = artificial intelligence, CECT = contrast-enhanced CT, CRC = colorectal cancer, CRLM = colorectal liver metastasis, FLL = focal liver lesion, PVP = portal venous phase

Of 514 patients with surgically confirmed CRC who underwent CECT for suspected CRLM, 221 were excluded for lacking reference standards/follow-up (*n* = 159), prior CRLM therapy (*n* = 33), inadequate PVP imaging (*n* = 18), or poor image quality (*n* = 11). An additional 15 were excluded because of software analysis failure, plus one case with multiple cysts indicative of biliary hamartoma (Fig. 3). Thus, 277 patients (182 men; median age, 70 years [range, 29–99]) constituted the final cohort. Of these, 212 had 989 true FLLs, including 324 CRLMs. The median FLL size was 6 mm (range, 1–86 mm), and the median CRLM size was 13 mm (range, 2–86 mm). Demographic details are presented in Table 1 and Supplement.

**Table 1:** Demographic and clinical characteristics of the study population

Variables	Values
Sex (male: female)	182:95
Age (y)*	71 (63, 78) in men 70 (62, 80) in women
Underlying cancer	
Colon cancer	180 (65.0)
Rectal cancer	97 (35.0)
Methods of FLL confirmation	
CT follow-up	126 (45.5)
Gadoxetic acid-enhanced MRI follow-up	32 (11.6)
Surgical resection	8 (2.9)
Gadoxetic acid-enhanced MRI and surgical resection	111 (40.1)
Preoperative chemotherapy (yes)	74 (26.7)
Diagnosis of FLLs (*n* = 1204)	
Hepatic cysts	593 (49.3)
Colorectal liver metastases	324 (26.9)
Hemangiomas	58 (4.8)
Hepatocellular carcinomas	4 (0.3)
Cholangiocarcinomas	2 (0.2)
Others†	8 (0.6)
Pseudolesions	215 (17.9)
FLL size	
< 5 mm	303 (30.6)
5–9 mm	401 (40.5)
10–19 mm	147 (14.9)
≥ 20 mm	138 (14.0)

Note—: Values are median (IQR) or number (percentage). FLL = focal liver lesion. *No difference between men and women (*P* = 0.98). †: chemotherapy associated focal hepatopathy (*n* = 3), focal hemorrhage (*n* = 2), focal venous dilatation (*n* = 1), fibrotic nodule (*n* = 1), regenerative nodule (*n* = 1)

### Performance of standalone AI for detecting all FLLs

The FOM of standalone AI was 0.54 (95% CI: 0.49, 0.58). The false positive rate and positive predictive value of AI were 18.1% (219/1208, 95% CI: 15.0, 21.8) and 84.5% (529/626, 95% CI: 79.1, 88.7%), respectively. The most common false positives detected by AI were prominent intrahepatic bile ducts (40.2%, 39/97) (Supplement). AI detection rates varied by lesion size, successfully identifying 85.3% of FLLs ≥ 10 mm, 58.9% of those 5–9 mm, and 16.5% of those < 5 mm (Table 2). For CRLMs, overall detection was 71.0%, with higher success (90.7%) for lesions ≥ 10 mm compared to 45.9% for lesions measuring 5–9 mm (Table 2).

**Table 2:** Focal liver lesion (FLL) detection and segmentation with AI depending on FLL size

FLL size	AI detection (A)	AI segmentation (B)	Radiologists* (C)	Difference [95% CI]		
				A vs. B	A vs. C	B vs. C
**All FLLs**						
< 5 mm	0.17 (50/303) [0.12, 0.22]	0.16 (48/303) [0.12, 0.22]	0.40 (725/1818) [0.35, 0.45]	-0.01 [-0.02, 0.01]	-0.23 [-0.29, -0.18]	-0.24 [-0.30, -0.18]
*P*-value				0.313	< 0.001	< 0.001
5–9 mm	0.59 (236/401) [0.53, 0.65]	0.53 (214/401) [0.48, 0.62]	0.60 (1437/2406) [0.56, 0.64]	-0.06 [-0.14, 0.03]	-0.01 [0.05, 0.04]	-0.06 [-0.15, 0.02]
*P*-value				0.217	0.706	0.145
≥ 10 mm	0.85 (243/285) [0.81, 0.89]	0.82 (234/285) [0.77, 0.86]	0.86 (1463/1710) [0.81, 0.89]	-0.03 [-0.06, -0.002]	-0.003 [-0.05, 0.04]	-0.04 [-0.09, 0.02]
*P*-value				0.030	0.890	0.188
**CRLM**						
< 5 mm	0.23 (5/22) [0.11, 0.41]	0.18 (4/22) [0.07, 0.40]	0.33 (43/132) [0.19, 0.50]	-0.05 [-0.12, 0.03]	-0.10 [-0.24, 0.04]	− 0.14 [-0.35, 0.06]
*P*-value				0.295	0.189	0.197
5–9 mm	0.46 (50/109) [0.32, 0.61]	0.35 (38/109) [0.22, 0.50]	0.48 (314/654) [0.39, 0.57]	-0.11 [-0.29, 0.07]	-0.02 [-0.12, 0.07]	-0.13 [-0.28, 0.02]
*P*-value				0.228	0.659	0.098
≥ 10 mm	0.91 (175/193) [0.85, 0.94]	0.87 (168/193) [0.81, 0.92]	0.89 (1035/1158) [0.85, 0.93]	-0.04 [-0.08, 0.002]	0.01 [-0.03, 0.06]	-0.02 [-0.08, 0.04]
*P*-value				0.056	0.567	0.425

Note—. Per-lesion analysis. Data are pooled results across six readers (e.g., 989 ⋅ 6 = 5934 lesions). Data in parentheses are numerators/denominators; data in brackets are 95% CIs. AI = artificial intelligence, CRLM = colorectal liver metastasis. *In the review session where participants were requested to detect all FLLs. The presented data is a pooled analysis result of six radiologists. *P*-value < 0.05 indicates a statistically significant difference

In addition to detecting FLLs, the AI software provided automatic segmentation. Segmentation accuracy similarly depended on lesion size, with correct segmentation rates of 82.1% (234/285) for lesions ≥ 10 mm, 53.4% (214/401) for lesions 5–9 mm, and 15.8% (48/303) for lesions < 5 mm. For CRLMs, segmentation success was 87.0% (168/193) for lesions ≥ 10 mm and 34.9% (38/109) for lesions 5–9 mm, indicating better AI segmentation performance for larger lesions.

### Comparison of the performance of standalone AI and radiologists alone for detecting all FLLs

**Fig. 4:**
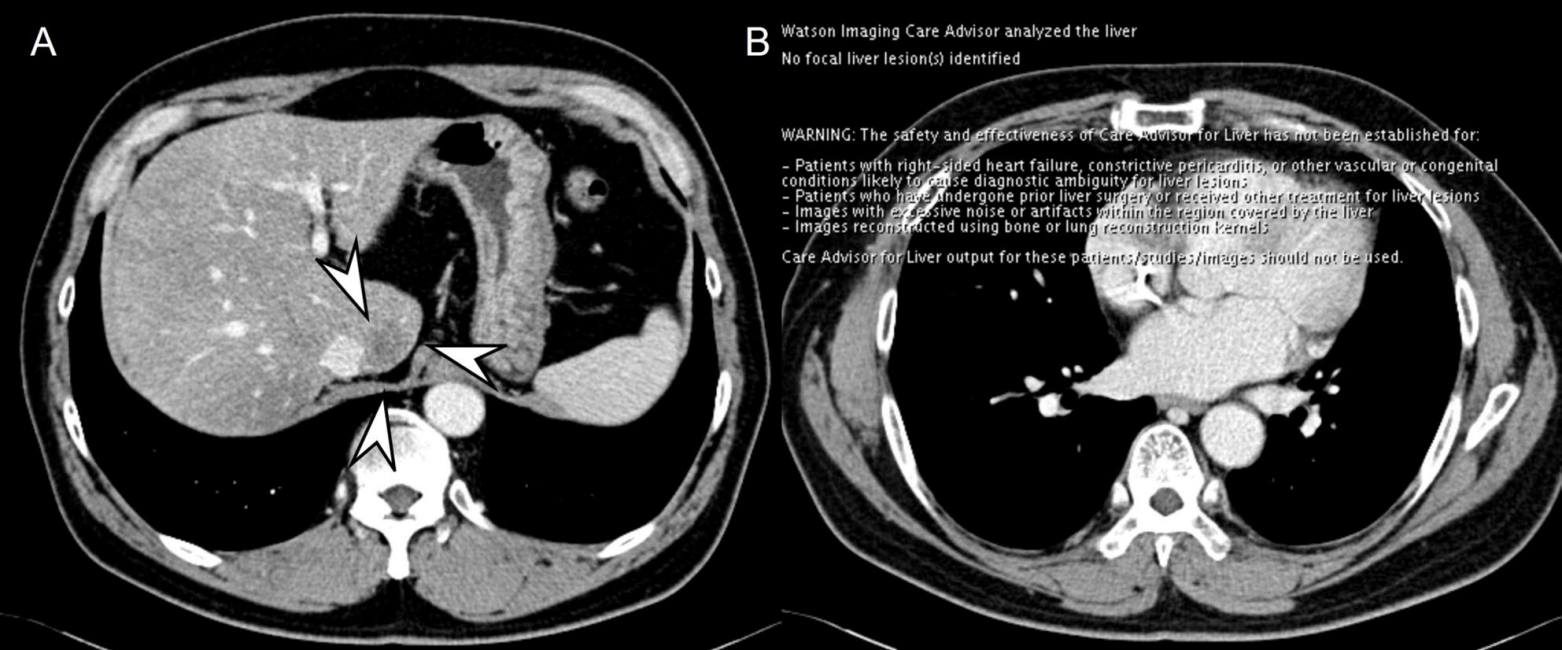
A 52-year-old male patient with surgically confirmed colorectal liver metastasis (CRLM). On portal venous phase (**A**), 20 mm CRLM is observed in segment 1 (arrowheads) while no focal liver lesions are identified on AI software (**B**)

**Fig. 5:**
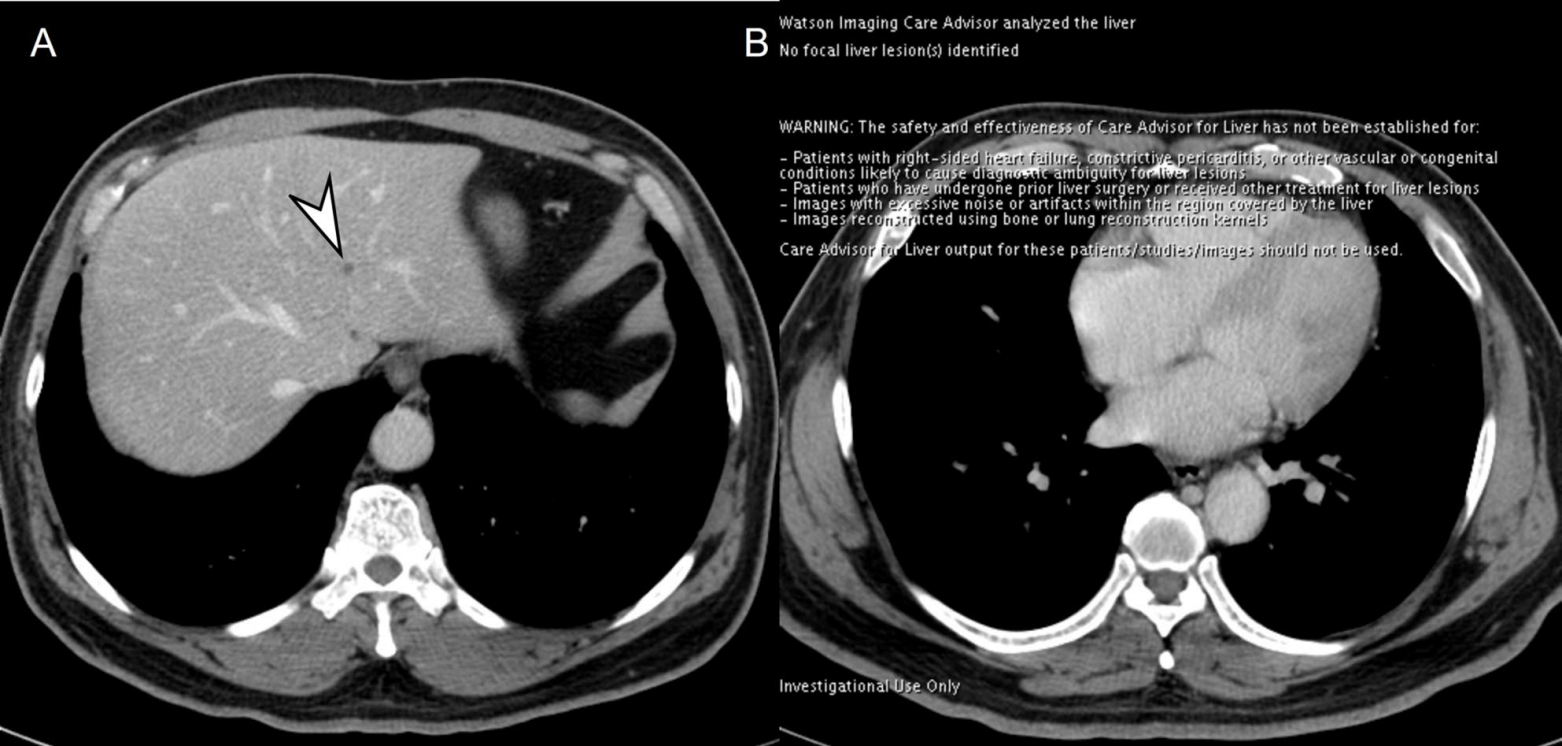
A 71-year-old male patient with hepatic cyst. On portal venous phase (**A**), a 5 mm hepatic cyst is visualized in liver left lateral segment (arrowhead). All reviewers detected it as definitely or probably not metastasis, while no focal liver lesions were identified on AI software (**B**)

**Fig. 6:**
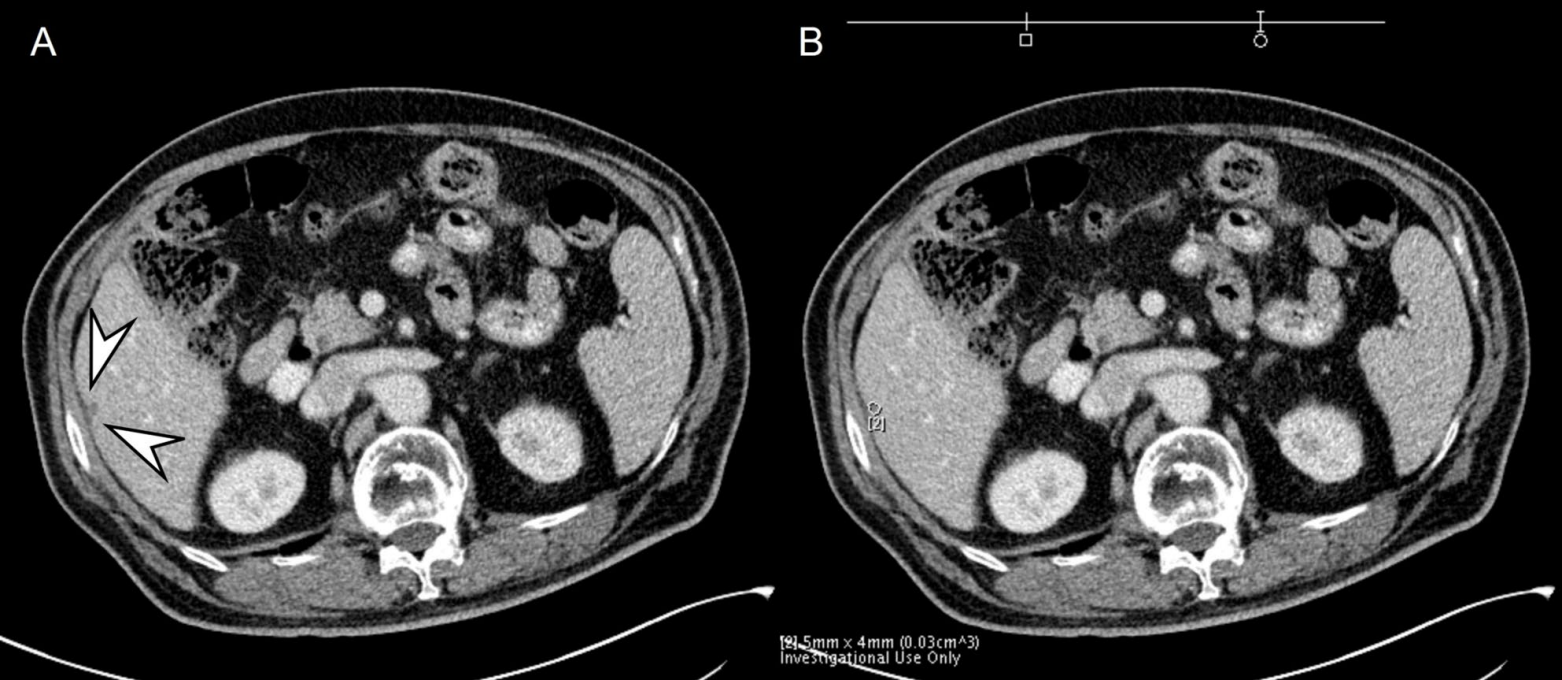
A 69-year-old male patient with surgically confirmed colorectal liver metastasis (CRLM). On portal venous phase (**A**), 5 mm CRLM is seen in the segment 5 subcapsular area (arrowheads), which is correctly detected and segmented with AI software (**B**). In the review session of radiologists alone, half (3/6) reviewers missed the CRLM while 5 out of 6 reviewers reported it in the session with AI

The pooled FOM of radiologists alone was 0.61 (95% CI: 0.58, 0.64), which was higher than the FOM of AI alone regardless of the appropriateness of the segmentation (*P* < 0.001 for both) (Fig. 4). In FLLs < 5 mm, radiologists showed a higher FOM (0.40, 95% CI: 0.35, 0.45) than standalone AI (*P* < 0.001 for both) (Fig. 5) while no difference was observed in FLLs 5–9 mm and ≥ 10 mm (FOM: 0.60 [95% CI: 0.56, 0.64] and 0.86 [95% CI: 0.81, 0.89], respectively). There was no difference in FOM between standalone AI and radiologists in CRLMs regardless of the size (Table 2; Fig. 6).

### Comparison between the FLL detection rates by radiologists with and without AI in sessions reporting all FLLs

The detection rates of CRLM were 0.78 (95% CI: 0.73, 0.83) for radiologists alone and 0.79 (95% CI: 0.76, 0.82) for radiologists aided by AI (*P* = 0.405). No difference was observed between the review sessions with and without AI among the senior reviewers (0.78 [95% CI: 0.71, 0.85] vs. 0.78 [0.64, 0.91], *P* = 0.724) and junior reviewers (0.80 [95% CI: 0.75, 0.84] vs. 0.79 [0.71, 0.86], *P* = 0.317) (Table 3).

**Table 3:** Comparison between the detection rates of radiologists requested to detect all FLLs with and without AI

Reviewers	Radiologists	Radiologists with AI	Difference	*P*-value
All FLL				
All radiologists (*n* = 6)	0.61 (3625/5934) [0.58, 0.64]	0.61 (3626/5934) [0.58, 0.64]	0.00 [-0.02, 0.02]	0.986
Senior radiologists (*n* = 3)	0.58 (1711/2967) [0.55, 0.60]	0.60 (1784/2967) [0.57, 0.63]	0.03 [0.004, 0.05]	0.022
Junior radiologists (*n* = 3)	0.65 (1914/2967) [0.61, 0.68]	0.62 (2842/2967)) [0.59, 0.65]	-0.02 [-0.05, -0.002]	0.029
CRLM				
All radiologists (*n* = 6)	0.78 (1392/1944) [0.73, 0.83]	0.79 (1392/1944) [0.76, 0.82]	0.01 [-0.02, 0.03]	0.405
Senior radiologists (*n* = 3)	0.78 (679/972) [0.64, 0.91]	0.78 (688/972) [0.71, 0.85]	0.01 [-0.07, 0.09]	0.724
Junior radiologists (*n* = 3)	0.79 (713/972) [0.71, 0.86]	0.80 (704/972) [0.75, 0.84]	0.01 [-0.02, 0.04]	0.317

Note—. Per-lesion analysis. Data are pooled results across six readers (e.g., 989 ⋅ 6 = 5934 lesions). Data in parentheses are numerators/denominators; data in brackets are 95% CIs. *P*-value < 0.05 indicates a statistically significant difference between the sessions of radiologists alone and radiologists with AI. FLL = focal liver lesion, AI = artificial intelligence, CRLM = colorectal liver metastasis

The radiologists’ performance in diagnosing CRLM was not different in sessions with and without AI, in terms of sensitivity (67.8% vs. 68.0%, respectively, *P* = 0.924), specificity (83.9% vs. 82.8%, respectively, *P* = 0.289) and accuracy (78.6% vs. 77.9%, respectively, *P* = 0.358) (Table 4). No difference in sensitivity, specificity, PPV, NPV and accuracy was observed in per-patient analysis (Table S2).

**Table 4:** Comparison between diagnostic performance for CRLM of radiologists requested to detect all FLLs with and without AI

Review sessions	Sensitivity (%)	Specificity (%)	PPV (%)	NPV (%)	Accuracy (%)
Requested to detect all FLLs					
Radiologists	68.0 (1321/1944) [61.7, 73.6]	82.8 (3302/3990) [79.6, 85.5]	65.8 (1321/2009) [59.0, 72.0]	84.1 (3302/3925) [77.7, 88.9]	77.9 (4623/5934) [74.2, 81.2]
Radiologists with AI	67.8 (1319/1944) [61.3,73.8]	83.9 (3348/3990) [81.1, 86.4]	67.3 (1319/1961) [60.6, 73.3]	84.3 (3348/3973) [78.0, 89.0]	78.6 (4667/5934) [75.2, 81.7]
Difference (%)	-0.1 [-2.2, 2.0]	1.2 [-1.0, 3.3]	1.5 [-1.4, 4.4]	0.1 [-0.8, 1.1]	0.7 [-0.8, 2.3]
*P*-value	0.924	0.289	0.309	0.768	0.359
Requested to report only FLLs with suspicion of CRLM					
Radiologists	62.5 (1215/1944) [55.6, 68.9]	89.8 (3584/3990) [87.5, 91.8]	75.0 (1215/1621) [69.0, 80.1]	83.1 (3584/4313) [76.7, 88.0]	80.9 (4799/5934) [76.8, 84.4]
Radiologists with AI	66.8 (1299/1944) [59.7, 73.2]	89.6 (3574/3990) [87.1, 91.6]	75.7 (1299/1715) [70.1, 80.6]	84.7 (3574/4219) [78.5, 89.4]	82.1 (4873/5934) [78.3, 85.4]
Difference (%)	4.3 [2.1, 6.5]	-0.3 [-1.7, 1.2]	0.8 [-1.9, 3.4]	1.6 [0.7, 2.5]	1.2 [0.0, 2.5]
*P*-value	< 0.001	0.730	0.560	< 0.001	0.046

Note—. Per-lesion analysis. Data are pooled results across six readers (e.g., 989 ⋅ 6 = 5934 lesions). Data in parentheses are numerators/denominators; data in brackets are 95% CIs. *P*-value < 0.05 indicates a statistically significant difference between the sessions of radiologists alone and radiologists with AI. CRLM = colorectal liver metastasis, FLL = focal liver lesion, AI = artificial intelligence, PPV = positive predictive value, NPV = negative predictive value

### Comparison of the FLL detection rates by radiologists with and without AI in sessions reporting only FLLs with suspicion of CRLM

The FOMs of CRLM detection were 0.65 (95% CI: 0.62, 0.68) without AI and 0.66 (95% CI: 0.63, 0.68) with AI (*P* = 0.099) (Table S3). The sensitivity for diagnosing CRLM was higher in the session with AI (66.8% [95% CI: 59.7, 73.2] vs. % 62.5% [55.6, 68.9], *P* < 0.001) while no difference of specificity was observed (89.6% [95% CI: 87.1, 91.6] vs. 89.8% [87.5, 91.8], *P* = 0.730). Accuracy marginally improved with AI (82.1% [95% CI: 78.3, 85.4] vs. 80.9% [76.8, 84.4], *P* = 0.046) (Table 4). Per-patient analysis also demonstrated higher sensitivity with AI assistance (92.7% vs. 90.5%, *P* = 0.013, Table S2).

### Comparison of the reading time between sessions with and without AI

In the sessions reporting all FLLs, the median reading time decreased from 38.0 s (95% CI: 37.0, 38.0) to 30.0 s (95% CI: 28.0, 32.0) after applying AI (*P* < 0.001) (Table 5, Table S4). In the sessions reporting only FLLs with suspicion of CRLM, the median reading time was 17.0 s (95% CI: 16.0, 18.4) and 18.0 s (95% CI: 17.0, 19.0) with and without AI, respectively (*P* < 0.001).

**Table 5:** Comparison between the reading time of radiologists with and without AI

Reviewers	Radiologists (s)	Radiologists with AI (s)	Difference*	*P*-value*
Requested to detect all FLLs				
All radiologists (*n* = 6)	38.0 (21.0, 67.0)	30.0 (15.0, 53.0)	-8.5 [-9.5, -7.5]	< 0.001
Senior radiologists (*n* = 3)	29.0 (17.0, 54.5)	24.0 (12.0, 45.0)	-5.0 [-6.5, -4.0]	< 0.001
Junior radiologists (*n* = 3)	50.0 (28.0, 78.5)	35.0 (21.0, 58.5)	-12.5 [-14.0, -11.0]	< 0.001
Difference†	16.0 [9.0, 22.0]	9.0 [4.0, 14.0]	–	–
*P*-value†	< 0.001	< 0.001	–	–
Requested to report only FLLs with suspicion of CRLM				
All radiologists (*n* = 6)	18.0 (9.0, 43.0)	17.0 (9.0, 34.0)	-2.5 [-3.0, -2.0]	< 0.001
Senior radiologists (*n* = 3)	10.0 (7.0, 19.0)	12.0 (8.0, 20.0)	0.5 [0.0, 0.5]	0.038
Junior radiologists (*n* = 3)	38.0 (15.0, 71.0)	29.0 (13.0, 49.0)	-10.0 [-11.5, -8.0]	< 0.001
Difference†	24.0 [18.0, 31.0]	14.0 [11.0, 18.0]	–	–
*P*-value†	< 0.001	< 0.001	–	–

Note—. Data are presented as median (interquartile range); data in brackets are 95% CIs. *†: *P*-value < 0.05 indicates a statistically significant difference *between the sessions of radiologists alone and radiologists with AI and †between senior and junior radiologists in the same session. FLL = focal liver lesion, CRLM = colorectal liver metastasis

AI assistance reduced the reading time for both junior radiologists (median, 50.0 s [95% CI: 46.0, 52.0] vs. 35.0 s [33.0, 38.0], *P* < 0.001) and senior radiologists (median, 29.0 s [95% CI: 28.0, 31.0] vs. 24.0 s [22.0, 27.0], *P* < 0.001) in the sessions reporting all FLLs. Junior radiologists showed longer reading time than senior radiologists, and the gap decreased from 16.0 s to 9.0 s when AI was applied (Table 5). In the sessions reporting only FLLs with suspicion of CRLM, the median reading time decreased from 38.0 s (95% CI: 35.0, 43.0) to 29.0 s (95% CI: 27.0, 31.0) for the junior radiologists (*P* < 0.001), while the reading time increased from 10 s (95% CI: 9.0, 11.0) to 12 s (95% CI: 11.0, 12.0) for the senior radiologists (*P* = 0.038). The reading time gap between senior and junior radiologists decreased from 24.0 s to 14.0 s when AI was applied.

### The impact of the scope of reporting on the detection and diagnosis of CRLM

Without AI, both radiologists’ detection rate and sensitivity for CRLM decreased (*P* < 0.001 for all) in the session reporting only FLLs with suspicion of CRLM compared with the session reporting all FLLs (Table S5). With AI, the session reporting only FLLs with suspicion of CRLM still showed a lower detection rate (*P* < 0.001), but there was no significant difference in sensitivity (*P* = 0.315) compared with the session which all FLLs were reported (Table S5).

### Impact of clinical experience on CRLM diagnosis in sessions with and without AI

In the review session without AI, junior radiologists showed higher sensitivity than senior radiologists (65.1% [95% CI: 58.7, 71.1] vs. 59.9% [52.3, 67.1], *P* = 0.001) when reporting only FLLs with suspicion of CRLM. However, the senior radiologists showed higher accuracy than the junior radiologists (84.1% [95% CI: 79.6, 87.7] vs. 77.7% [73.7, 81.2], *P* < 0.001) due to low specificity in junior radiologists (83.8% [95% CI: 80.3, 86.8] vs. 95.8% [94.1, 97.1], *P* < 0.001) (Table S6). It was the same in sessions with AI: senior radiologists showed higher accuracy (84.8% [95% CI: 87.0, 88.2] vs. 79.4% [75.5, 82.8], *P* < 0.001) owing to higher specificity of senior radiologists (94.7% [95% CI: 92.8, 96.1] vs. 84.5% [80.9, 87.5], *P* < 0.001) (Table S7).

## Discussion

Detecting CRLM remains challenging due to the subtle contrast difference on CT. To address this issue, attempts have been made to use AI, similar to its application in chest radiography or mammography, for pancreatic lesions and prostate cancers [10–17]. However, Yet, few studies have assessed the impact of AI on radiologists for FLL detection [4, 6]. In our study, standalone AI (DUO-L) detected 71.0% of CRLMs overall, with better performance in lesions ≥ 10 mm (85.3%) but limited efficacy in those < 5 mm. Although AI alone did not surpass radiologists for detection, it served as a valuable adjunct: when readers focused only on suspicious CRLMs, AI increased sensitivity without reducing specificity. It also reduced overall reading time, suggesting potential workflow benefits in oncologic surveillance by decreasing missed metastases and expediting interpretation.

Our data suggest that “satisfaction of search” may influence radiologists’ performance without AI, as interpreting only suspected CRLMs led to lower sensitivity in the absence of AI. The AI support helped offset this effect, even though free-response FOM measures between sessions with and without AI were similar. The boost in sensitivity likely stems from AI prompts that highlight possible metastases, mitigating oversights once an obvious lesion is identified [3, 18]. This finding aligns with previous research showing that perceptual errors contribute significantly to missed diagnoses in radiology [18], and suggests that AI assistance may help mitigate these cognitive biases.

The clinical implications of our findings extend beyond improving detection rates. The reduction in reading time with AI assistance—particularly for reporting all FLLs—could significantly impact workflow efficiency in busy oncologic imaging practices. AI assistance significantly reduced reading time in sessions requiring complete FLL reporting for both junior and senior radiologists. Furthermore, junior radiologists required longer reading time than senior radiologists in every reading session, but this gap decreased in sessions with AI assistance. With the growing burden of imaging studies and increasing complexity of cancer follow-up protocols, time-saving tools that maintain or improve diagnostic accuracy represent valuable additions to clinical practice. Moreover, the differential impact of AI based on radiologist experience suggests potential applications in training and education, particularly for less experienced readers who may benefit more substantially from AI support. AI’s impact on reading time varied by radiologist experience and the scope of interpretation tasks. For junior radiologists, AI consistently reduced reading time across all tasks. However, for senior radiologists focusing solely on suspicious CRLM, the median reading time slightly increased from 10 to 12 s with AI (*P* = 0.038). This modest increase might reflect the additional cognitive load of reviewing both original and AI-labeled images, which may offset efficiency gains for highly experienced readers with already rapid interpretation times. This observation suggests that AI implementation strategies may need to be tailored based on radiologist experience and specific clinical tasks.

Although AI correctly detected or segmented over 80% of FLLs ≥ 10 mm, its overall FLL detection rates were lower than some earlier reports [3, 4, 6], likely because our cohort included many smaller lesions (median 6 mm). This finding aligns with earlier studies showing that both human readers and AI perform worse on small FLLs [3, 6, 12]. The limited AI performance for lesions under 10 mm or occult cancers is well-documented in chest, breast, and liver imaging [5, 6, 19, 20]. The lower detection rate for small lesions reflects an inherent trade-off between sensitivity and specificity in AI algorithm design; lowering the detection threshold would capture more small lesions but substantially increase false positives, potentially impairing clinical workflow. Moreover, determining an optimal detection threshold is even harder in liver CT because of low signal-to-noise ratios and partial volume averaging artifacts in small FLLs. Notably, the most common false positives in our study were prominent intrahepatic bile ducts (40.2%), which was also a major source of false positive in a recent MRI study [21]. This likely reflects the AI algorithm’s response to their tubular hypoattenuating appearance on portal venous phase images, which may mimic the imaging characteristics of hypovascular lesions. However, these false positives are readily identifiable by radiologists based on their characteristic anatomical location adjacent to portal vein branches and their tubular morphology on contiguous images. This observation underscores the complementary nature of AI-assisted detection: while AI provides systematic coverage to minimize oversight, radiologist expertise remains essential for contextual interpretation and false-positive dismissal.

Our results differ from a previous study reporting that AI detected 92.7% of CRLMs identified by radiologists and 53.7% of CRLMs overlooked by radiologists [3]. The discrepancy may stem from our higher proportion of < 5 mm FLLs and different methodologies (their use of radiologic reports vs. our direct review). While their study used radiologic reports as the reference standard, we employed direct image review by expert radiologists and pathologic confirmation, providing a more rigorous assessment of both AI and radiologist performance against a clinically relevant gold standard.

Although AI reduced the reading time and increased readers’ sensitivity without compromising specificity in our study, it did not affect aspects of performance related to readers’ experience. Specifically, senior and junior radiologists showed different patterns. The senior radiologists showed higher accuracy, higher specificity, higher PPV, lower sensitivity, and shorter reading time than their junior counterparts. The junior radiologists detected more FLLs in a longer reading time, but they had lower accuracy in their classification. This pattern suggests that AI, in its current implementation, supplements human reading without fundamentally altering the underlying differences in diagnostic approaches between radiologists of varying experience levels. As suggested by previous research, search and classification errors appear to be largely independent [22], highlighting the need for more sophisticated AI algorithms that can address both error types to reduce experience-related performance gaps [4]. Beyond technical performance, the integration of AI into clinical workflows raises important questions about how radiologists interact with these tools. Our study design, which directly compared reading sessions with and without AI, provides valuable insights into this human-AI interaction. The improvement in sensitivity without loss of specificity when using AI suggests that radiologists appropriately incorporated AI input rather than over-relying on it, a critical consideration for successful clinical deployment of AI tools.

Our study has several limitations that should be considered when interpreting the results. First, its single-center, retrospective design may have introduced bias. Additionally, the exclusion of patients without adequate reference standards may have introduced selection bias, and our cohort may not fully represent the spectrum of cases encountered in routine clinical practice. Second, the absence of time constraints for readers deviates from routine clinical practice, where time pressure may influence both AI and human performance differently. Third, our focus solely on FLLs, without assessing extrahepatic lesions or prior images, simplifies the interpretation task compared to real-world practice. Fourth, only PVP scans were used, since the AI model accepts that phase exclusively, but multiphase evaluation is standard in clinical practice. The software’s strict phase requirements excluded 15 cases deemed PVP by human reviewers, highlighting a practical challenge in AI implementation. Future research should address these limitations through prospective, multi-center studies incorporating multiphase imaging. Fifth, we did not use DICE scores to assess segmentation accuracy. This is because our study focused on detection performance rather than segmentation quality, and we only evaluated clinically relevant mis-segmentations that could affect lesion measurement or characterization. The AI software is primarily designed to aid detection and reduce reading time by automatically measuring FLL size, rather than to provide precise segmentation for volumetric analysis. Sixth, although our study demonstrated statistically significant improvements in sensitivity with AI assistance, the overlapping confidence intervals suggest modest effect sizes. Future studies with larger sample sizes and prospective designs are warranted to confirm these findings and further define the clinical benefit of AI-assisted detection in CRLM surveillance. Lastly, we did not investigate the potential impact of CT scanner specifications, including differences between older and modern multi-detector CT systems, on AI or radiologist performance.

In conclusion, AI software may improve radiologists’ performance by increasing the sensitivity of diagnosing CRLM on CECT, without decreasing specificity, and reducing the reading time. While standalone AI does not yet match experienced radiologists’ performance, its value as an assistive tool—particularly for less experienced readers and in high-volume settings—demonstrates the potential of human-AI collaboration in oncologic imaging.

## Supplementary Information

Below is the link to the electronic supplementary material.

Supplementary Material 1

## Data Availability

The datasets used and/or analysed during the current study are available from the corresponding author on reasonable request.

## Abbreviations

AI: Artificial intelligence
CECT: Contrast-enhanced CT
CRC: Colorectal cancer
CRLM: Colorectal liver metastasis
FLL: Focal liver lesion
FOM: Figure-of-merit
PVP: Portal venous phase

## References

[1] Morgan E, Arnold M, Gini A, Lorenzoni V, Cabasag CJ, Laversanne M, et al. Global burden of colorectal cancer in 2020 and 2040: incidence and mortality estimates from GLOBOCAN. Gut. 2023;72:338–44. 36604116 10.1136/gutjnl-2022-327736

[2] Rompianesi G, Pegoraro F, Ceresa CD, Montalti R, Troisi RI. Artificial intelligence in the diagnosis and management of colorectal cancer liver metastases. World J Gastroenterol. 2022;28:108–22. 35125822 10.3748/wjg.v28.i1.108 PMC8793013

[3] Nakai H, Sakamoto R, Kakigi T, Coeur C, Isoda H, Nakamoto Y. Artificial intelligence-powered software detected more than half of the liver metastases overlooked by radiologists on contrast-enhanced CT. Eur J Radiol. 2023;163:110823. 37059006 10.1016/j.ejrad.2023.110823

[4] Ying H, Liu X, Zhang M, Ren Y, Zhen S, Wang X, et al. A multicenter clinical AI system study for detection and diagnosis of focal liver lesions. Nat Commun. 2024;15:1131. 38326351 10.1038/s41467-024-45325-9 PMC10850133

[5] Kim DW, Lee G, Kim SY, Ahn G, Lee J-G, Lee SS, et al. Deep learning–based algorithm to detect primary hepatic malignancy in multiphase CT of patients at high risk for HCC. Eur Radiol. 2021;31:7047–57. 33738600 10.1007/s00330-021-07803-2

[6] Kim K, Kim S, Han K, Bae H, Shin J, Lim JS. Diagnostic performance of deep Learning-Based lesion detection algorithm in CT for detecting hepatic metastasis from colorectal cancer. Korean J Radiol. 2021;22:912–21. 33686820 10.3348/kjr.2020.0447 PMC8154788

[7] Finlay IG, Meek D, Brunton F, McArdle CS. Growth rate of hepatic metastases in colorectal carcinoma. Br J Surg. 1988;75:641–4. 3416116 10.1002/bjs.1800750707

[8] Tanaka K, Shimada H, Miura M, Fujii Y, Yamaguchi S, Endo I, et al. Metastatic tumor doubling time: most important prehepatectomy predictor of survival and nonrecurrence of hepatic colorectal cancer metastasis. World J Surg. 2004;28:263–70. 14961200 10.1007/s00268-003-7088-3

[9] Ronneberger O, Fischer P, Brox T. U-Net: convolutional networks for biomedical image segmentation. In: Navab N, Hornegger J, Wells WM, Frangi AF, editors. Medical image computing and Computer-Assisted Intervention – MICCAI 2015. Cham: Springer International Publishing; 2015. pp. 234–41.

[10] Bilello M, Gokturk SB, Desser T, Napel S, Jeffrey RB, Beaulieu CF. Automatic detection and classification of hypodense hepatic lesions on contrast-enhanced venous-phase CT. Med Phys. 2004;31:2584–93. 15487741 10.1118/1.1782674

[11] Chen Q, Zhu Y, Chen Y, Wang F, Hu X, Ye Y, et al. Applicability of multidimensional convolutional neural networks on automated detection of diverse focal liver lesions in multiphase CT images. Med Phys. 2023;50:2872–83. 36441108 10.1002/mp.16140

[12] Cheng C-T, Cai J, Teng W, Zheng Y, Huang Y-T, Wang Y-C, et al. A flexible three-dimensional heterophase computed tomography hepatocellular carcinoma detection algorithm for generalizable and practical screening. Hepatol Commun. 2022;6:2901–13. 35852311 10.1002/hep4.2029 PMC9512477

[13] Szeskin A, Rochman S, Weiss S, Lederman R, Sosna J, Joskowicz L. Liver lesion changes analysis in longitudinal CECT scans by simultaneous deep learning voxel classification with SimU-Net. Med Image Anal. 2023;83:102675. 36334393 10.1016/j.media.2022.102675

[14] Vorontsov E, Cerny M, Régnier P, Di Jorio L, Pal CJ, Lapointe R, et al. Deep learning for automated segmentation of liver lesions at CT in patients with colorectal cancer liver metastases. Radiol Artif Intell. 2019;1:180014. 33937787 10.1148/ryai.2019180014 PMC8017429

[15] Zhou J, Wang W, Lei B, Ge W, Huang Y, Zhang L, et al. Automatic detection and classification of focal liver lesions based on deep convolutional neural networks: A preliminary study. Front Oncol. 2020;10:581210. 33585197 10.3389/fonc.2020.581210 PMC7878526

[16] Abi Nader C, Vetil R, Wood LK, Rohe M-M, Bône A, Karteszi H, et al. Automatic detection of pancreatic lesions and main pancreatic duct dilatation on portal venous CT scans using deep learning. Invest Radiol. 2023;58:791–8. 37289274 10.1097/RLI.0000000000000992

[17] Roest C, Yakar D, Rener Sitar DI, Bosma JS, Rouw DB, Fransen SJ, et al. Multimodal AI combining clinical and imaging inputs improves prostate cancer detection. Invest Radiol. 2024;59:854–60. 39074400 10.1097/RLI.0000000000001102

[18] Bruno MA, Walker EA, Abujudeh HH. Understanding and confronting our mistakes: the epidemiology of error in radiology and strategies for error reduction. Radiographics. 2015;35:1668–76. 26466178 10.1148/rg.2015150023

[19] Nam JG, Park S, Hwang EJ, Lee JH, Jin K-N, Lim KY, et al. Development and validation of deep Learning-based automatic detection algorithm for malignant pulmonary nodules on chest radiographs. Radiology. 2019;290:218–28. 30251934 10.1148/radiol.2018180237

[20] Lee SE, Han K, Yoon JH, Youk JH, Kim E-K. Depiction of breast cancers on digital mammograms by artificial intelligence-based computer-assisted diagnosis according to cancer characteristics. Eur Radiol. 2022;32:7400–8. 35499564 10.1007/s00330-022-08718-2

[21] Zhang L, Wang L, Zhang Y, Zhang X, Huang Y, Zheng C, et al. Fully automated multi-sequence detection and alignment of focal liver lesions in dynamic contrast-enhanced MRI. Eur Radiol; 2025. online a head of print. 10.1007/s00330-025-12055-5 41175201

[22] Hsieh SS, Inoue A, Yalon M, Cook DA, Gong H, Sudhir Pillai P, et al. Targeted training reduces search errors but not classification errors for hepatic metastasis detection at Contrast-Enhanced CT. Acad Radiol. 2024;31:448–56. 37567818 10.1016/j.acra.2023.06.017 PMC10853479

