# Supplementary material for: Evaluation of the performance of radiologists assisted by AI in detecting colorectal liver metastases on contrast-enhanced CT
Source: Cancer Imaging. 2026 Feb 16;26:43. doi: 10.1186/s40644-026-00998-x (PMC13014994; doi:10.1186/s40644-026-00998-x)
Supplement: Supplementary file 1 — Supplementary Material 1 [file 40644_2026_998_MOESM1_ESM.docx]

**SUPPLEMENTARY MATERIALS AND METHODS**

**CT Acquisition**

Contrast-enhanced CT (CECT) scans were acquired using multiple multi-detector CT scanners from different vendors (Definition, Definition AS, Definition AS+, Definition Flash, Emotion 16, Sensation 10, Sensation 16, Sensation 64, Sensation Cardiac 64, Volume Zoom, Siemens Healthcare; HiSpeed, LightSpeed 16, LightSpeed Pro16, LightSpeed Plus, LightSpeed VCT, LightSpeed Ultra, Discovery CT750 HD, GE HealthCare; Mx8000, Mx8000 Dual, Brilliance 64, Philips Healthcare; Aquilion, Aquilion One, Canon Medical Systems). Patents underwent CT exams using scanners with 2- (n = 1), 4- (n = 30), 10- (n = 9), 16- (n = 85), 64- (n = 122), 128- (n = 27), 256- (n = 1) and 320- channel (n = 2) detector configurations. CT scans were performed with 120–140 kVp and tube-current modulation if available for a given scanner. A standard dose of iodinated contrast agent (Iopromide 370, 1.5 mL/kg or Iobitridol 350, 1.6 mL/kg) was administered at a rate of 3–4 mL/s, followed by a 20-mL saline flush.

**Artificial intelligence (AI) model development**

The development database consisted of 4580 abdominal CECT studies from 3357 patients, collected across 7 institutions in North America, Europe, and Asia. A total of 19384 focal liver lesions (FLLs) visible in axial portal phase were segmented in 3D by one of 32 annotating radiologists with at least 3 years of experience in reading abdominal CECT. Among the development dataset, 740 examinations of 649 patients with 2767 FLLs were used for internal testing. Detailed information of development dataset is in **Table S1**.

Two deep networks with 2.5D U-Nets architectures, taking respectively 3 and 9 slices as inputs, were trained to segment all visible FLLs in axial portal phase. Model architectures are given in **Figure S1**. The 3-slice model relies on a DenseNet-161 network to encode 192x192x3 crops around the liver, while the 9-slice model relies on a DenseNet-121 encoder to process 512x512x9 inputs without any pre-cropping. Encoders were initialized with ImageNet pre-trained weights, and the decoders using the He method. The first model is trained with the focal Tversky and binary cross-entropy losses, while the second model is trained with a custom Tversky loss that penalizes false positives only in slices without ground truth lesions and the mean square error loss in a deep supervision fashion. The Adam optimizer with an initial learning rate of 0.0001 and the Tensorflow 1.15 library were leveraged for both models. Best model weights were selected using the validation set. At inference, the predictions of both models are averaged, in an ensembling fashion.

The rest of the algorithmic pipeline also included pre-filtering modules aimed at rejecting series that would not contain at least one third of the liver axial extension or non-portal phase series, and post-processing modules aimed at refining the lesion contours, identifying and removing false detections, and measuring the lesion. More details can be found in a patent application publication of US20220270254A[1]. Note that the portal phase was chosen as the unique required input for the developed algorithm in order to maximize its applicability in various clinical scenarios.

**Identification of focal liver lesions**

The presence or absence of focal liver lesions (FLLs) was determined by follow-up CECT (n = 126), follow-up MRI (n = 32), or hepatic resection (n = 119) within 8 weeks of the index CECT examination. All patients who received hepatic resection had either gadoxetic acid-enhanced liver MRI (n = 111) or four-phase liver CT (n = 8). The median interval between the index CECT and follow-up MRI was 12 days (range, 1–58 days), the median interval between the index CECT and the earliest follow-up CECT was 40 days (range, 1–57 days), and the median interval between CECT and hepatic resection was 16 days (range, 1–51 days).

In patients with follow-up CECT, the median interval between the last follow-up CECT and the index CECT was 1800 days (range, 43–4208 days), and their earliest follow-up CECT was used as a reference standard for identifying number of FLLs, while the last CECT was used to characterize the identified FLLs. Newly developed metastases within 3 months in non-surgical patients were defined as occult metastases and missed FLLs on the index CECT. Among the 126 patients who underwent follow-up CECT, two patients had metastasis (n = 2), and the interval between the index and follow-up CECT examinations was 43 and 52 days. In 124 patients without metastasis, the last CECT examinations at 3–5 months of follow-up (n = 72, median interval 91 days), 6–12 months of follow-up (n = 38, median interval 236 days), or more than 2 years of follow-up (n = 14, median 1792 days) were used to characterize non-metastatic FLLs. Serum markers such as Carcinoembryonic antigen or Carbohydrate antigen 19-9 were not used because the stability of such tumor markers did not always guarantee stable disease or disease regression.

**Diagnosis of focal liver lesions**

All malignant tumors were histologically confirmed within 8 weeks of CECT. A clinical diagnosis was made for benign FLLs according to the following imaging features and stability of size or size decrease on follow-up.

*Hepatic cysts—* Hepatic cysts were defined as round, ovoid, or lobulated nodules showing no enhancement on dynamic phases of CECT or gadoxetic acid-enhanced MRI. In addition, marked hyperintensity on heavily T2-weighted image and low attenuation (0–15 HU) on CECT without enhancement were used to diagnose hepatic cysts [2].

*Hemangiomas—* Hemangiomas were clinically diagnosed based on their characteristic features, including a peripheral discontinuous nodular enhancement pattern on CECT and gadoxetic acid-enhanced MRI without significant interval change during follow-up, or with marked hyperintensity on T2-weighted imaging [3].

*Focal venous dilatation—* Focal venous dilatation was defined as aneurysmal dilatation of the hepatic vein with an intrahepatic veno-venal shunt, which showed the same enhancement pattern of the hepatic vein on follow-up CECT and was stable during follow-up.

*Chemotherapy-induced focal hepatopathy—* A clinical diagnosis was made based on ill-defined margin with T1 isointensity, fuzzy arterial phase hyperenhancement, and a poorly defined hepatobiliary phase defect without diffusion restriction, and its resolution on follow-up imaging in patients who received chemotherapy [4].

*Pseudolesions (False positive lesions) —* Pseudolesions were defined as unseen FLLs on follow-up imaging, normal anatomic structures, or hemodynamic changes such as focal fat deposition or sparing if one of the reviewers or artificial intelligence software detected them as FLLs.

**SUPPLEMENTARY RESULTS**

**Size and diagnosis of focal liver lesions**

There were 989 FLLs in 212 patients. Malignancies accounted for 33.4% (330/989), including colorectal liver metastases (CRLMs, n = 324), hepatocellular carcinoma (HCC, n = 4), intrahepatic cholangiocarcinomas (iCCAs, n = 2). The median size of malignant FLLs was 13 mm (range 2–86 mm) in CRLMs and 34 mm (range 11–64 mm) in HCCs. The sizes of the two iCCAs were 44 mm and 45 mm. All malignancies were histologically confirmed.

In the remaining 659 benign FLLs, hepatic cysts were the most common (n = 593), followed by hemangioma (n = 58), chemotherapy-induced focal hepatopathy (n = 3), focal hemorrhagic hepatopathy (n = 2), focal venous dilatation (n = 1), regenerative nodule (n = 1), and fibrotic nodule (n = 1). Among them, four benign FLLs were surgically confirmed—a fibrotic nodule (2 mm), two cases of focal hemorrhagic hepatopathy (9 mm, 23 mm), and a regenerative nodule (12 mm) —while the others were clinically diagnosed. The median size of benign FLLs was 5 mm (range, 1–46 mm) in hepatic cysts, 9 mm (range: 3–63 mm) in hemangiomas, 10 mm in chemotherapy-induced focal hepatopathy. The size of focal venous dilatation was 10 mm.

**False positive lesions detected by AI software**

A total of 97 FLLs detected by AI software were determined to be pseudolesions. The most common pseudolesions detected by AI were prominent intrahepatic bile ducts (40.2%, 39/97), focal fat deposition or hemodynamic changes resulting from hepatic third flow (20.6%, 20/97), pseudolesions generated by incomplete segmentation (17.5%, 17/97), normal anatomical structures such as vessels or diaphragm (n = 16), pseudolesions caused by a partial volume averaging artifact (n = 3), and attenuation changes being invisible on MRI (n = 2).

**Reviewer experience in CRLM diagnosis**

The interactions between CRLM diagnosis and reviewers’ clinical experience (senior and junior) were tested using the interaction terms in the generalized estimating equations. In sessions without AI assistance, the clinical experience and the impact of the scope had relationship in terms of specificity and positive predictive value (PPV) and accuracy (*P* <0.001 for all) while insignificant in sensitivity (*P* = 0.43) and negative predictive value (NPV, *P* = 0.59). In sessions with AI assistance, the clinical experience and the impact of the scope had relationship in terms of specificity and PPV (*P* <0.001 for both) and accuracy (*P* = 0.01) while insignificant in sensitivity (*P* = 0.75) and NPV (*P* = 0.83).

**SUPPLEMENTARY REFERENCES**

1. Palma G, Fernandez P, Dufort P, et al. Lesion detection artificial intelligence pipeline computing system. https://ppubs.uspto.gov/dirsearch-public/print/downloadBasicPdf/20220270254?requestToken=eyJzdWIiOiJhNGU2NzhjYS1lNGQ1LTQ4YzMtYjkyYi04OGYyOGU0NzI3NDUiLCJ2ZXIiOiIxODE0NDk0Yi03YzRkLTQxZWMtYTg1Zi01NDMxYzIwYWJkOGQiLCJleHAiOjB9 Filed May 10, 2022. Published Aug 25, 2022. Accessed September 19, 2024.

2. Borhani AA, Wiant A, Heller MT. Cystic hepatic lesions: a review and an algorithmic approach. *Am J Roentgenol* 2014;203:1192–1204

3. Fowler KJ. Universal Liver Imaging Lexicon: Imaging Atlas for Research and Clinical Practice. *RadioGraphics* 2023;43(1):e220066

4. You S-H, Park BJ, Kim YH. Hepatic Lesions that Mimic Metastasis on Radiological Imaging during Chemotherapy for Gastrointestinal Malignancy: Recent Updates. *Korean J Radiol* 2017;18:413–426

**Table S1. Development database**

|  | **Patients** | **Examinations** | **Focal liver lesions (FLLs)** |
| --- | --- | --- | --- |
| **Sample size** | 3357 | 4580 | 19384 |
| **Sample for internal testing** | 649 (19.3) | 740 (16.2) | 2767 (14.3) |
| **Cirrhosis (yes)** | 800 (23.8) | 1040 (22.7) | 2993 (15.4) |
| **FLLs (yes)** | 2705 (80.1) | 3480 (76.0) | 19384 (100.0) |
| **Diagnosis of FLLs** | | | |
| Benign low-attenuating FLLs (cyst or lipoma) | 1021 (30.4) | 1178 (25.7) | 4011 (20.7) |
| Hepatocellular carcinoma | 560 (16.7) | 636 (13.9) | 1194 (6.2) |
| Liver metastasis | 394 (11.7) | 614 (13.4) | 8404 (43.4) |
| Hemangioma | 420 (12.5) | 473 (10.3) | 735 (3.8) |
| Granuloma | 285 (8.5) | 322 (7.0) | 1492 (7.7) |
| Cholangiocarcinoma | 114 (3.4) | 147 (3.2) | 201 (1.0) |
| **FLL size** | | | |
| < 5 mm | 156 (4.6) | 168 (3.7) | 468 (2.4) |
| 5–9 mm | 982 (29.3) | 1183 (25.8) | 4680 (24.1) |
| 10–20 mm | 841 (25.1) | 1111 (24.3) | 7923 (40.9) |
| > 20 mm | 726 (21.6) | 1018 (22.2) | 6313 (32.6) |

Note—. Data are number (percentage).

**Table S2. Per-patient based comparison between diagnostic performance for CRLM of radiologists requested to detect all FLLs with and without AI**

| **Review sessions** | **Sensitivity (%)** | **Specificity (%)** | **PPV (%)** | **NPV (%)** | **Accuracy (%)** |
| --- | --- | --- | --- | --- | --- |
| **Requested to detect all FLLs** | | | | | |
| **Radiologists** | 94.3(713/756) [92.4, 95.8] | 82.3 (746/906) [79.7, 84.7] | 81.7 (713/873) [79.0, 84.1] | 94.6 (746/789) [92.7, 95.9] | 87.8 (1459/1662) [86.1, 89.3] |
| **Radiologists with AI** | 94.2 (712/756) [92.3, 95.6] | 82.3 (746/906) [79.7, 84.7] | 81.7 (712/872) [78.9, 84.1] | 94.4 (746/790) [92.6, 95.8] | 87.7 (1458/1662) [86.1, 89.2] |
| **Difference (%)** | -0.1 [-1.7, 1.4] | 0.0 [-2.2, 2.2] | 0.0 [-0.4, 0.4] | 0.0 [-0.5, 0.4] | -0.1 [-1.5, 1.3] |
| ***P*-value** | 0.866 | >0.999 | 0.976 | 0.841 | 0.933 |
| **Requested to report only FLLs with suspicion of CRLM** | | | | | |
| **Radiologists** | 90.5 (684/756) [88.2, 92.4] | 86.2 (781/906) [83.8, 88.3] | 84.5 (684/809) [81.9, 86.9] | 91.6 (781/853) [89.5, 93.2] | 88.1 (1465/1662) [86.5, 89.6] |
| **Radiologists with AI** | 92.7 (701/756) [90.6, 94.4] | 85.8 (777/906) [83.3, 87.9] | 84.5 (701/830) [81.8, 86.8] | 93.4 (777/832) [91.5, 94.9] | 88.9 (1478/1662) [87.3, 90.3] |
| **Difference (%)** | **2.2** [0.5, 4.0] | -0.4 [-2.4, 1.5] | 0.0 [-0.4, 0.4] | 0.6 [0.2, 1.1] | 0.8 [-0.6, 2.1] |
| ***P*-value** | 0.013 | 0.663 | 0.984 | 0.005 | 0.256 |

Note—. Data are pooled results across six readers (e.g., 277 × 6 = 1662 patients) Data in parentheses are numerators/denominators; data in brackets are 95% CIs. *P*-value < 0.05 indicates a statistically significant difference between the sessions of radiologists alone and radiologists with AI. CRLM = colorectal liver metastasis, FLL = focal liver lesion, PPV = positive predictive value, NPV = negative predictive value.

**Table S3. Comparison between the detection rates of radiologists requested to report only FLLs with suspicion of CRLM with and without AI**

| **Reviewers** | **Radiologists** | **Radiologists with AI** | **Difference** | ***P*-value** |
| --- | --- | --- | --- | --- |
| **All FLL (n = 989)** | | | | |
| **All radiologists (n= 6)** | 0.27 (1626/5934) [0.24, 0.31] | 0.29 (1717/5934) [0.25, 0.33] | 0.02 [0.003, 0.03] | 0.013 |
| **Senior radiologists (n =3)** | 0.22 (666/2967) [0.19, 0.27] | 0.25 (735/2967) [0.21, 0.29] | 0.02 [0.01, 0.04] | <0.001 |
| **Junior radiologists (n = 3)** | 0.32 (960/2967) [0.28, 0.37] | 0.33 (982/2967) [0.29,0.38] | 0.01 [-0.01, 0.03] | 0.420 |
| **CRLM (n = 324)** | | | | |
| **All radiologists (n= 6)** | 0.65 (1217/1944) [0.62, 0.68] | 0.66 (1300/1944) [0.63, 0.68] | 0.01 [-0.002, 0.02] | 0.099 |
| **Senior radiologists (n =3)** | 0.64 (582/972) [0.61, 0.66] | 0.64 (629/972) [0.62, 0.67] | 0.01 [-0.01, 0.02] | 0.242 |
| **Junior radiologists (n =3)** | 0.66 (635/972) [0.64, 0.69] | 0.67 (972/671) [0.65, 0.70] | 0.01 [-0.01, 0.02] | 0.177 |

Note—. Per-lesion analysis. Data are pooled results across six readers (e.g., 989 × 6 = 5934 lesions). Data in parentheses are numerators/denominators; data in brackets are 95% CIs. *P*-value <0.05 indicates a statistically significant difference between the sessions of radiologists alone and radiologists with AI. FLL = focal liver lesions, CRLM = colorectal liver metastasis, AI = artificial intelligence.

**Table S4. Linear mixed-effects model for review time comparison between sessions with and without AI assistance**

| **Effect** | **Coefficient (s)** | ***P*-value** |
| --- | --- | --- |
| **Requested to detect all FLLs** | | |
| **AI (with and without AI assistance)** | -15.0 [-20.1, -10.0] | <0.001 |
| **Experience (senior vs. junior)** | -21.6 [-40.0, -3.2] | 0.021 |
| **AI and experience interaction** | 12.2 [5.0, 19.3] | 0.001 |
| **Requested to report only FLLs with suspicion of CRLM** | | |
| **AI (with and without AI assistance)** | -14.7 [-18.1, -11.3] | <0.001 |
| **Experience (senior vs. junior)** | -37.5 [-68.1, -6.9] | 0.016 |
| **AI and experience interaction** | 15.5 [10.8, 20.3] | <0.001 |

Note—. Data in brackets are 95% CI. Coefficients are expressed in seconds. *P*-value <0.05 indicates a statistically significant effect. FLL = focal liver lesion, AI = artificial intelligence, CRLM = colorectal liver metastasis.

**Table S5.** **Impact of the scope in six radiologists’ performance to detect CRLM in sessions with and without AI assistance**

| **Variables** | **Reporting all FLLs** | **Reporting only FLLs with suspicion of CRLMs** | **Difference** | ***P*-value** |
| --- | --- | --- | --- | --- |
| **Without AI assistance** | | | | |
| **FOM** | 0.78 (1392/1944) [0.73, 0.83] | 0.65 (1217/1944) [0.62, 0.68] | -0.1 [-0.2, -0.1] | <0.001 |
| **Sensitivity (%)** | 68.0 (1321/1944) [61.7,73.6] | 62.5 (1215/1944) [55.6, 68.9] | -5.5 [-8.0, -3.0] | <0.001 |
| **Specificity (%)** | 82.8 (3302/3990) [79.6, 85.5] | 89.8 (3584/3990) [87.5, 91.8] | 7.1 [5.0, 9.0] | <0.001 |
| **Accuracy (%)** | 77.9 (4623/5934) [74.2, 81.2] | 89.0 (4799/5934) [76.8, 84.4] | 3.0 [1.0, 5.0] | <0.001 |
| **With AI assistance** | | | | |
| **FOM** | 0.79 (1392/1944) [0.76, 0.82] | 0.66 (1300/1944) [0.63, 0.68] | -0.1 [-0.2, -0.1] | <0.001 |
| **Sensitivity (%)** | 67.8 (1319/1944) [61.3, 73.8] | 66.8 (1299/1944) [59.7, 73.2] | -1.0 [-3.0, 1.0] | 0.315 |
| **Specificity (%)** | 83.9 (3348/3990) [81.1, 86.4] | 89.6 (3574/3990) [87.1, 91.6] | 5.7 [4.1, 7.2] | <0.001 |
| **Accuracy (%)** | 78.6 (4667/5934) [75.2, 81.7] | 82.1 (4873/5934) [78.3, 85.4] | 3.5 [2.2, 4.8] | <0.001 |

Note—. Per-lesion analysis. Data are pooled results across six readers (e.g., 989 × 6 = 5934 lesions). Data in parentheses are numerators/denominators; data in brackets are 95% CIs. *P*-value <0.05 indicates a statistically significant difference between the sessions of radiologists alone and radiologists with AI. CRLM = colorectal liver metastasis, AI = artificial intelligence, FLL = focal liver lesions, FOM = figure-of-merit.

**Table S6.** **Impact of clinical experience on CRLM diagnosis**

| **Review sessions** | **Sensitivity (%)** | **Specificity (%)** | **PPV (%)** | **NPV (%)** | **Accuracy (%)** |
| --- | --- | --- | --- | --- | --- |
| **Requested to detect all FLLs** | | | | | |
| **Senior radiologists** | 66.4 (645/972)  [59.7, 72.4] | 85.3 (1701/1995) [82.5, 87.7] | 68.7 (645/939)  [61.8, 74.8] | 83.9 (1701/2028)  [77.3, 88.8] | 79.1 (2346/2967) [75.2, 82.4] |
| **Junior radiologists** | 69.5 (676/972)  [63.5, 75.0] | 80.3 (1601/1995)  [76.1, 83.9] | 63.2 (676/1070)  [56.0, 69.8] | 84.4 (1601/1897)  [78.1, 89.1] | 76.7 (2277/2967)  [72.9, 82.0] |
| **Difference (%)** | 3.2 [0.7, 5.7] | -5.0 [-7.8, -2.2] | -5.5 [-8.8, -2.2] | 0.5 [-0.5, 1.6] | -2.3 [-4.3, -0.4] |
| ***P*-value** | 0.013 | <0.001 | 0.001 | 0.337 | 0.020 |
| **Requested to report only FLLs with suspicion of CRLM** | | | | | |
| **Senior radiologists** | 59.9 (582/972)  [52.3, 67.1] | 95.8 (1912/1995) [94.1,97.1] | 87.5 (582/665)  [82.4,91.3] | 83.1 (1912/2302)  [76.7, 88.0] | 84.1 (2494/2967)  [79.6, 87.7] |
| **Junior radiologists** | 65.1 (633/972)  [58.7, 71.1] | 83.8 (1672/1995)  [83, 86.8] | 66.2 (633/956)  [59.6, 72.2] | 83.1 (1672/2011)  [76.5,88.2] | 77.7 (2305/2967)  [73.7, 81.2] |
| **Difference (%)** | 5.2 [2.1, 8.4] | -12.0 [-14.6, -9.4] | -21.3 [-25.6, -17.0] | 0.1 [-1.1, 1.3] | -6.4 [-8.4, -4.4] |
| ***P*-value** | 0.001 | <0.001 | <0.001 | 0.887 | <0.001 |

Note—. Per-lesion analysis. Data are pooled results across six readers (e.g., 989 × 6 = 5934 lesions). Data in parentheses are numerators/denominators; data in brackets are 95% CIs. *P*-value <0.05 indicates a statistically significant difference between senior and junior radiologists. CRLM = colorectal liver metastasis, PPV = positive predictive value, NPV = negative predictive value, FLL = focal liver lesions.

**Table S7. Impact of clinical experience on CRLM diagnosis with AI assistance**

| **Review sessions** | **Sensitivity (%)** | **Specificity (%)** | **PPV (%)** | **NPV (%)** | **Accuracy (%)** |
| --- | --- | --- | --- | --- | --- |
| **Requested to detect all FLLs** | | | | | |
| **Senior radiologists with AI** | 66.0 (642/972)  [59.5, 72.0] | 87.2 (1739/1995)  [84.9, 89.2] | 71.5 (642/898)  [65.1, 77.1] | 84.1 (1739/2069)  [77.8, 88.8] | 80.2 (2381/2967)  [76.7, 83.3] |
| **Junior radiologists with AI** | 69.7 (677/972)  [62.7, 75.8] | 80.7 (1609/1995)  [77.0, 83.9] | 63.7 (677/1063)  [56.5, 73.0] | 84.5 (1609/1904)  [78.2, 89.2] | 77.0 (2286/2967)  [73.4, 83.0] |
| **Difference (%)** | 3.6 [1.0, 6.2] | -6.5 [-8.7, -4.3] | -7.8 [-15.0, -5.1] | 0.5 [-0.7, 1.6] | -3.2 [-4.9, -1.5] |
| ***P*-value** | 0.006 | <0.001 | <0.001 | 0.437 | <0.001 |
| **Requested to report only FLLs with suspicion of CRLM** | | | | | |
| **Senior radiologists with AI** | 64.6 (628/972)  [57.0, 71.5] | 94.7 (1889/1995)  [92.8, 96.1] | 85.6 (628/734)  [80.6, 89.4] | 84.6 (1889/2233)  [78.5, 89.2] | 84.8 (2517/2967)  [80.7, 88.2] |
| **Junior radiologists with AI** | 69.0 (671/972)  [62.1, 75.2] | 84.5 (1685/1995)  [80.9, 87.5] | 68.4 (671/981)  [62.0, 74.2] | 84.8 (1685/1986)  [78.4, 89.6] | 79.4 (2356/2967)  [75.5, 82.8] |
| **Difference (%)** | 4.4 [1.6, 7.2] | -10.2 [-12.8, -7.6] | -17.2 [-21.2, -13.2] | 0.2 [-0.9, 1.4] | -5.4 [-7.4, -3.5] |
| ***P*-value** | 0.002 | <0.001 | <0.001 | 0.660 | <0.001 |

Note—. Per-lesion analysis. Data are pooled results across six readers (e.g., 989 × 6 = 5934 lesions). Data in parentheses are numerators/denominators; data in brackets are 95% CIs. *P*-value <0.05 indicates a statistically significant difference between senior and junior radiologists with AI. CRLM = colorectal liver metastasis, AI = artificial intelligence, PPV = positive predictive value, NPV = negative predictive value, FLL = focal liver lesions.

**Figure S1. Deep network architectures for lesion detection**
